# *Mycobacterium tuberculosis* Rv0580c Impedes the Intracellular Survival of Recombinant Mycobacteria, Manipulates the Cytokines, and Induces ER Stress and Apoptosis in Host Macrophages via NF-κB and p38/JNK Signaling

**DOI:** 10.3390/pathogens10020143

**Published:** 2021-02-01

**Authors:** Md Kaisar Ali, Lambert Nzungize, Khushnood Abbas, Nzaou Stech Anomene Eckzechel, M. A. Abo-kadoum, Ulrich Aymard Ekomi Moure, Mohammed Asaad, Aftab Alam, Junqi Xu, Jianping Xie

**Affiliations:** 1 Institute of Modern Biopharmaceuticals, State Key Laboratory Breeding Base of Eco-environment and Bio-resource of the Three Gorges Area, Key Laboratory of Eco-environments in Three Gorges Reservoir Region, Ministry of Education, School of Life Science, Southwest University, Beibei, Chongqing 400715, China; kaisarali@swu.edu.cn (M.K.A.); nzulapa@email.swu.edu.cn (L.N.); stechanomen@email.swu.edu.cn (N.S.A.E.); abokadoum@email.swu.edu.cn (M.A.A.-k.); aymard@email.swu.edu.cn (U.A.E.M.); mohasaad85@oiu.edu.sd (M.A.); jj20200901@swu.edu.cn (J.X.); 2 College of Animal Science and Technology, Southwest University, Beibei, Chongqing 400715, China; 3 School of Computer Science and Technology, Zhoukou Normal University, East Wenchang Street, Chuanhui District, Zhoukou City 466001, Henan Province, China; abbas@cigit.ac.cn; 4 Department of Botany and Microbiology, Faculty of Science, Al-Azhar University, Assuit Branch, Assuit 71524, Egypt; 5 Department of Biotechnology, Faculty of Science and Technology, Omdurman Islamic University, Omdurman, Khartoum 11111, Sudan; 6 Centre for Interdisciplinary Research in Basic Sciences, Jamia Millia Islamia, New Delhi 110025, India; aalam6@jmi.ac.in

**Keywords:** *Mycobacterium tuberculosis*, Rv0580c, cell wall, cytokines, apoptosis

## Abstract

The *Mycobacterium tuberculosis* (*M. tb*) genome encodes a large number of hypothetical proteins, which need to investigate their role in physiology, virulence, pathogenesis, and host interaction. To explore the role of hypothetical protein Rv0580c, we constructed the recombinant *Mycobacterium smegmatis* (*M. smegmatis*) strain, which expressed the Rv0580c protein heterologously. We observed that Rv0580c expressing *M. smegmatis* strain (Ms_Rv0580c) altered the colony morphology and increased the cell wall permeability, leading to this recombinant strain becoming susceptible to acidic stress, oxidative stress, cell wall-perturbing stress, and multiple antibiotics. The intracellular survival of Ms_Rv0580c was reduced in THP-1 macrophages. Ms_Rv0580c up-regulated the IFN-γ expression via NF-κB and JNK signaling, and down-regulated IL-10 expression via NF-κB signaling in THP-1 macrophages as compared to control. Moreover, Ms_Rv0580c up-regulated the expression of *HIF-1α* and ER stress marker genes via the NF-κB/JNK axis and JNK/p38 axis, respectively, and boosted the mitochondria-independent apoptosis in macrophages, which might be lead to eliminate the intracellular bacilli. This study explores the crucial role of Rv0580c protein in the physiology and novel host-pathogen interactions of mycobacteria.

## 1. Introduction

Tuberculosis (TB) remains a formidable global public health concern and leading cause of death in adults, as compare to other infectious diseases [1]. The Global TB report 2020 estimated that around 10 million people became sick with TB. Among these, around 1.2 million people died from TB occurred in HIV-negative individuals (1.7 million people died in 2000), and 208,000 people died from TB occurred in HIV-positive individuals (678,000 people died in 2000) [2]. Drug resistance makes this pathogen more deadly [1]. Great ambition and effective strategy are needed to produce a complete cure for this pathogen, which is still a challenging task. 

*M. tb* has coexisted with human hosts for thousands of years, and is the main cause of TB [3]. Upon contact with humans, *M. tb* reached in lower respiratory tract, where it phagocytosized by the macrophages [4]. Macrophages have evolved several defense tactics to eliminate the intracellular pathogens, such as *M. tb*. These defense tactics include acidic environment, generation of free radicals, restriction of essential nutrients access to the microbes, as well as secretion of anti-microbial peptides and cytokines, which are able to induce the protective host immune response, recruitment of other immune cells, and activate the suicidal activities such as autophagy or apoptosis [5]. Contrarily, *M. tb* evolved well-adapted counter-balance mechanisms against hostile environments and host immune defense strategies to survive, multiply and establish the infection inside the host [6,7]. The cell wall of *M. tb* is thick and made by unique moieties of lipids, glycolipids, lipoproteins and lypoglycans [8]. The *M. tb* cell wall serves as a defense against the host, by maintaining the neutral pH in macrophages [9]. The lipoprotein component of the *M. tb* cell wall induces the protective immune response [10]. Modification in the composition of the *M. tb* cell wall can be responsible for the considerable effect on several phenotypes, including the alteration of colony morphology and antibiotic resistance [11], and is directly associated with the virulence of *M. tb* [12]. For two decades, a large number of genomic and transcriptomic sequences of *M. tb* have been deposited in databases. However, about 27% of encoded proteins from the *M.tb* genome are yet to be explored their function, named as hypothetical protein [13,14]. Some of these hypothetical proteins have been characterized experimentally.

For example, Rv0079 is characterized as a DosR regulon which involves in the protein synthesis impediment [15], and capable to binds with toll-like receptors 2 to induce the secretion of cytokines [16]. The Rv3423.1 has been characterized as a histone acetyltransferase, and showed to boost the intracellular survival and multiplication of mycobacteria [17]. Rv2037c has been characterized as a phospholipase, which plays a vital role in mycobacterium cell-wall alterations, infection, increase the intracellular survival of bacilli, and induces the secretion of pro-inflammatory cytokines, such as IL-12 and TNFα [18]. Rv0177 is involved in the alteration of mycobacterium cell wall structure and regulate the cytokines secretion, which leads to induce the apoptosis in macrophages [19]. Rv2387 is associated with alterations in the mycobacterium cell wall and makes it more susceptible to the acidic conditions [11]. The putative suppressor *ΔRv3673c* mutant strain grows significantly faster, forms larger colonies, and is more sensitive to H_2_O_2_ as compared to the *ΔRv3673c* mutant strain [20]. Rv0580c was first identified and classified as cell wall/membrane protein, by using the DLC-MS/MS approach [21].

In this study, we first time explore the possible role of hypothetical protein Rv0580c in the physiology and host–pathogen interactions of *M. tb*. In the initial step toward exploring the role of this protein, we take benefit from the lack of *Rv0580c* gene in the *M. smegmatis* genome, a non-pathogenic strain, to construct two recombinant *M. smegmatis* strains. We cloned the *Rv0580c* gene from the *M. tb* H37Rv strain and expressed in the *M. smegmatis* mc^2^155 strain. We observed that Rv0580c could modify the colony morphology, and cell wall, in recombinant *M. smegmatis.* Moreover, our results also found that Rv0580c expressing *M. smegmatis* strain could modulate the cytokines profile and hypoxia factor of the THP-1 human macrophages, and induce unfolded protein response (UPR) and apoptosis.

## 2. Results

### 2.1. Rv0580c is Highly Conserved among Pathogenic Strains of Mycobacteria 

**Figure 1:**
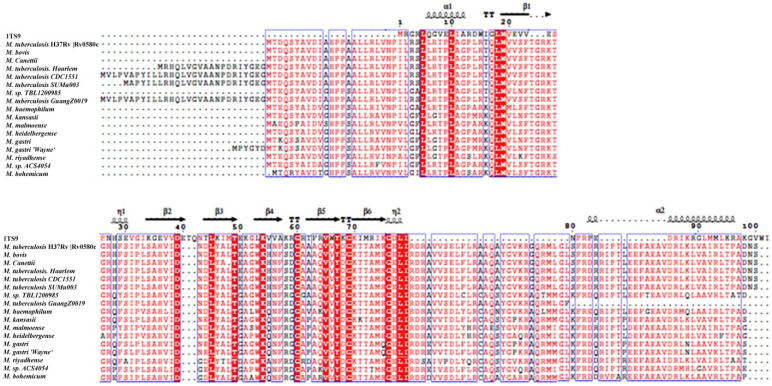
Rv0580c is conserved in pathogenic mycobacteria. Highly conserved residues are represented by the red color. The α-helix is represented by α, and the beta β-sheet is represented by β.

The BLASTp search indicated that the *M. tb* H37Rv Rv0580c protein sequence showed high similarity among the pathogenic strains of mycobacteria (Table S1). Genomic alignment of Rv0580c was predicted to encode a conserved protein. Structure-based sequence alignment with RNase P Protein (1TS9) analysis indicated that Rv0580c contains two α-helices and six β-sheets (Figure 1). 

### 2.2. Rv0580c can be Heterologously Expressed in M. smegmatis

**Figure 2:**
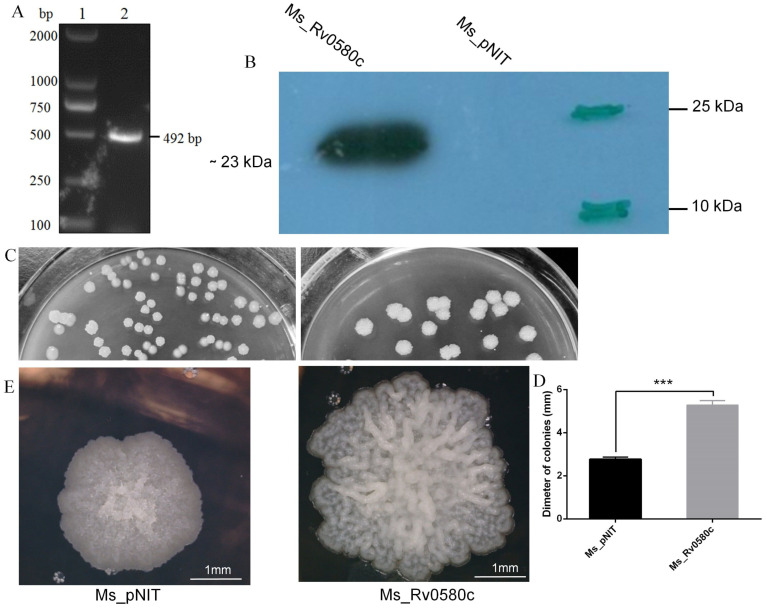
Heterologous expression of *M. tuberculosis* Rv0580c and its effect on cell surface architecture of *M. smegmatis*. (**A**) Amplification of *Rv0580*c gene from *M.tb* H37Rv by PCR; lane 1: DL2000; lane 2: Rv0580c. (**B**) Myc-tagged Rv0580c protein expression in *M. smegmatis* by applying mouse anti-myc antibody, demonstrated by Western blot. (**C**) Ms_pNIT and Ms_Rv0580c were grown in 7H10 agar solid medium supplemented with ε-caprolactam at 37 °C. Images were captured on day 5. (**D**) Diameters of the colonies were measured. (**E**) Ms_pNIT and Ms_Rv0580c colonies were analyzed by microscope and colony surface architectures were recorded. Data points represent the mean of three separate experiments, and error bars are +/− SEM. * *p* < 0.05, ** *p* < 0.01, and *** *p* < 0.001.

The Rv0580c gene was amplified from the *M. tb* H37Rv genome (Figure 2A) by using specific primers (Table S2). The 492 bp length of Rv0580c gene was was detected in Ms_Rv0580c strain. Western Blot confirmed that only the Ms_Rv0580c strain expressed ~23 kDa Rv0580c-myc protein, while it was absent in the Ms_pNIT strain (Figure 2B).

### 2.3. Rv0580c Modified the Colony Morphology of M. smegmatis

To understand the impact of Rv0580c on mycobacterial physiology, the Ms_pNIT and Ms_Rv0580c strains were grown on 7H10 agar plates supplemented with 0.2% (*w*/*v*) glucose, 0.2% (*v*/*v*) glycerol and 0.05% (*v*/*v*) tween 80. Plates were incubated at 37 °C for 4–5 days. We observed significantly larger sized colonies of Ms_Rv0580c as compared to Ms_pNIT, confirmed by the measurement of the diameter of a single colony (Figure 2C,D). Microscopic observation of single colony detected the dramatic changes in colony structure, by rough and wrinkled colony surface of Ms_Rv0580c, and smooth and non-wrinkled colony surface of Ms pNIT (Figure 2E). These observations suggested that Rv0580c modified the colony morphology of *M. smegmatis*. 

### 2.4. Rv0580c Increased the Susceptibility of M. smegmatis to Multiple Stresses and Antibiotics

**Figure 3:**
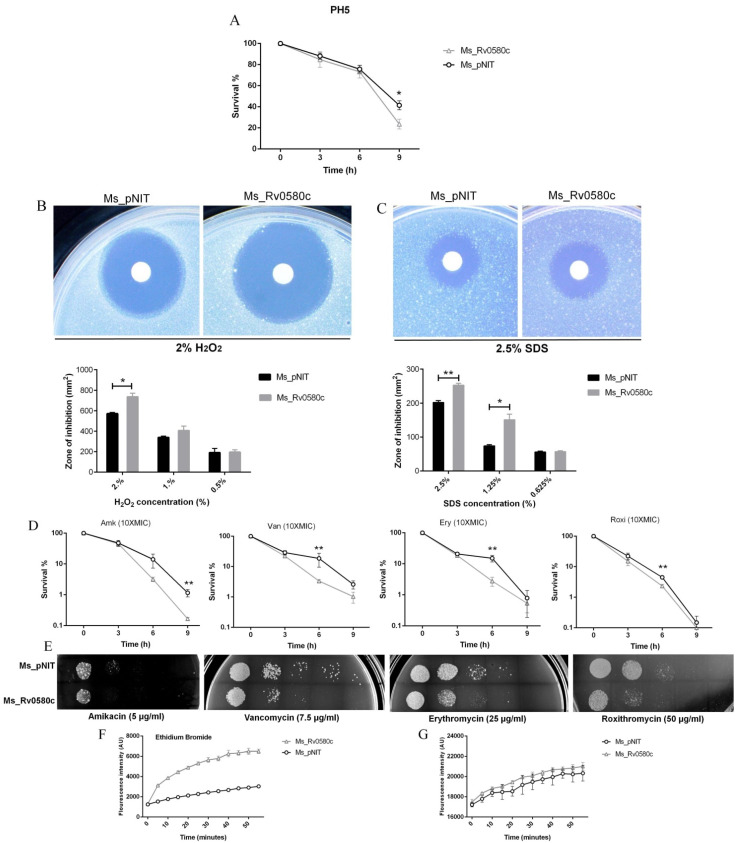
Effect of Rv0580c on multiple stresses, antibiotics and cell wall permeability in *M. smegmatis*. (**A**) The survival of in-vitro growing of log-phase culture of Ms_pNIT and Ms_Rv0580c in low pH (pH 5) 7H9 liquid medium. The log-phase culture of Ms_pNIT and Ms_Rv0580c were prepared as mentioned in the methods and treated with different concentrations of (**B**) H_2_O_2_ (**C**) and SDS. The zone of inhibition area was calculated after 3–4 days of incubation. (**D**) *Invitro* growths of Ms_pNIT and Ms_Rv0580c were exposed with 10 ×MIC of amikacin, vancomycin, erythromycin and roxithromycin then 10-fold serial dilution of bacteria were spotted on MB 7H9, and the bacterial number was counted after 3 days’ cultivation. (**E**) Ten-fold serial diluted Ms_Rv0580c and Ms_pNIT were spotted on 7H9 media containing amikacin (5 µg/mL), vancomycin (7.5 µg/mL), erythromycin (25 µg/mL) and roxithromycin (50 µg/mL). 7H9 medium was used as a control. All the plates were incubated for 3 days at 37 °C, and results were observed. (**F**) The Ms_pNIT and Ms_Rv0580c were treated with 2 μg/mL ethidium bromide (G) and 20 µM Nile red. The relative fluorescence unit of the intracellular ethidium bromide and Nile red were measured at 5 min intervals. Data points represent the mean of three separate experiments, and error bars are +/− SEM. * *p* < 0.05, ** *p* < 0.01, and *** *p* < 0.001.

Alteration in colony morphology might be because of cell wall modification, which might influence the response of mycobacteria to the antibiotics and multiple stresses that bacilli encounter inside the macrophages. Thus, we investigated the survival ability of Ms_Rv0580c to the antibiotics and multiple stress conditions that imitate the intracellular hostile environment. When both strains were incubated in low pH (pH 5) condition, the survival rate of Ms_Rv0580c significantly declined at the 9 h time point as compared to Ms_pNIT (Figure 3A). Both recombinant strains were treated with different concentrations of H_2_O_2_ (0.5%, 1.0% and 2.0%), to mimic the oxidative stress. At 2% H_2_O_2_ concentration, Ms_Rv0580c showed a significantly larger area zone of inhibition than Ms_pNIT, indicating that Ms_Rv0580c was more sensitive to the H_2_O_2_ as compared to control (Figure 3B). The sodium dodecyl sulfate (SDS) is a well-known anionic surfactant [22] that is used to generate cell surface stress [23]. Upon exposed with different concentrations of SDS (0.625%, 1.25% and 2.5%), Ms_Rv0580c showed a significantly larger zone of inhibition as compared to Ms_pNIT, indicating that Ms_Rv0580c was more sensitive to the cell surface stress than Ms_pNIT (Figure 3C). To investigate the effect of antibiotics, firstly we determined the minimum inhibitory concentration (MIC) of amikacin, vancomycin, erythromycin and roxithromycin for Ms_Rv0580c and Ms_pNIT (Table S3). To investigate the sensitivity of these antibiotics, we evaluated the survival of Ms_Rv0580c and Ms_pNIT upon exposure with 10 × MIC concentration of amikacin, vancomycin, erythromycin and roxithromycin. Results showed that Ms_Rv0580c confirmed more sensitivity to the amikacin, vancomycin, erythromycin and roxithromycin, as compared to Ms_pNIT (Figure 3D). These results were further verified by the spot cultures of Ms_Rv0580c and Ms_pNIT on 7H10 plates added with amikacin, vancomycin, erythromycin and roxithromycin, separately (Figure 3E). Collectively, these results indicate that Rv0580c increase the sensitivity of *M. smegmatis* to the stresses that mycobacteria face within macrophages.

### 2.5. Rv0580c Altered the Permeability of M. smegmatis Cell Wall

To determine whether the susceptibility to the multiple stresses and antibiotics altered the cell wall permeability, we performed the dye influx/accumulation assay of the polar compounds Nile red (NR) and Ethidium bromide (EtBr) [24]. We observed that the accumulation rate of EtBr was rapid and higher in Ms_Rv0580c as compared to Ms_pNIT (Figure 3F), while no difference was observed in the rate of NR accumulation (Figure 3G). These results suggested that Rv0580c increased the sensitivity of *M. smegmatis* to the multiple stresses and antibiotics, by increasing its cell wall permeability. 

### 2.6. Intracellular Survival of Ms_Rv0580c was Reduced

**Figure 4:**
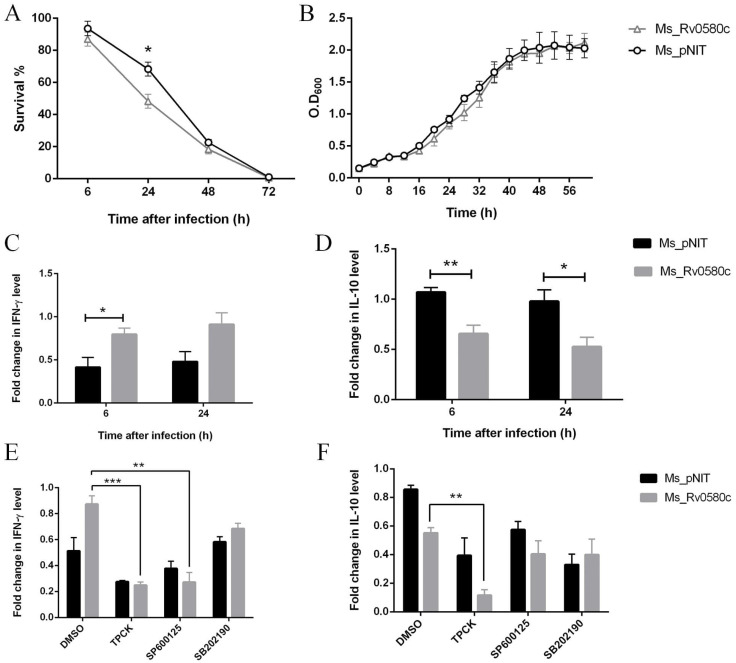
Rv0580c alters intracellular survival and expression of cytokines in *M. smegmatis* infected macrophages. (**A**) THP-1 macrophage cells were infected with Ms_pNIT and Ms_Rv0580c, as described in the materials and methods section. After 3–4 days of incubation, survival percentages were computed. (**B**) In-vitro growth kinetics of Ms_pNIT and Ms_Rv0580c in 7H9 liquid medium supplemented with ε-caprolactam (1%, *w*/*v*), tween 80 (0.05%, *v*/*v*) and kanamycin (100 μg/mL), determined at 37 °C by OD_600_ at 4 h interval. (**C**) PMA-differentiated THP-1 cells were infected with Ms_pNIT and Ms_Rv0580c. Total RNAs were collected from THP-1 cells at 6 h and 24 h post-infection, and carried out the RT-PCR to analyze the transcription levels of IFN-γ, (**D**) and IL-10. The TPCK (NF-κB inhibitor), SP600125 (JNK inhibitor) and SB202190 (p38 inhibitor) pre-treated THP-1 macrophage cells were infected with Ms_pNIT and Ms_Rv0580c. Total RNAs were isolated at 24 h post-infection, and carried out the RT-PCR to detect the transcription levels of (**E**) IFN-γ (**F**) and IL-10. DMSO was used as a control. Data points represent the mean of three separate experiments, and error bars are +/− SEM. * *p* < 0.05, ** *p* < 0.01, and *** *p* < 0.001.

At the initial stage of host interaction, *M. tb* interacts with macrophages, and intracellular survival of *M. tb* in macrophages is primarily stage of infection [25]. To investigate whether Rv0580c influences intracellular survival of mycobacterium inside host macrophages, Ms_pNIT and Ms_Rv0580c were used to infect the THP-1 human macrophages at a MOI of 10. The intracellular growth of bacteria in macrophages was analyzed by tallying the CFUs at different time points post-infection. Our results showed that the survival of Ms_Rv0580c in THP-1 cells was significantly reduced at 24 h post-infection as compared with Ms_pNIT, while no survival differences were detected at further time points (Figure 4A). Moreover, when we performed the in-vitro growth kinetics of Ms_Rv0580c and Ms_pNIT, no significant difference was observed (Figure 4B). These observations indicate that Rv0580c reduced the intracellular survival of bacilli. 

### 2.7. Ms_Rv0580c Modulated the Cytokines Profile of Macrophages Via NF-κB and JNK Signaling

During *M. tb* infection, cytokine secretion is a considerable part of regulating the inflammatory response and outcomes of infection in the host [26]. We have analyzed the expression level of cytokines from Ms_pNIT and Ms_Rv0580c infected THP-1 macrophage cells, at different time points. We have detected that IFN-γ expression was up-regulated (Figure 4C), while IL-10 expression was down-regulated (Figure 4D) in Ms_Rv0580c infected THP-1 cells, as compared to Ms_pNIT infected THP-1 cells, at 6 h and 24 h post-infection. 

To check whether intracellular signaling pathways were involved in the regulation IFN-γ and IL-10 in Ms_Rv0580c infected macrophages, we treated the differentiated THP-1 macrophage cells with N-tosyl-L-phenylalanine chloromethyl ketone (TPCK) (NF-κB inhibitor) [27], SP600125 (JNK inhibitor) [28] and SB202190 (p38 inhibitor) [29], for 1 h before infection. Our results showed that the IFN-γ expression was abolished, after treatment with NF-κB and JNK inhibitors in Ms_Rv0580c infected THP-1 cells, as compared to Ms_pNIT infected THP-1 cells, at 24 h post-infection (Figure 4E). However, the IL-10 expression was increased after treatment with only NF-κB inhibitor in Ms_Rv0580c infected THP-1 cells, as compared to Ms_pNIT infected THP-1 cells, at 24 h post-infection (Figure 4F). Taken together, these data suggested that Ms_Rv0580c up-regulated IFN-γ via the NF-κB and JNK signaling pathways, while down-regulating IL-10 via the NF-κB signaling pathway in macrophages.

### 2.8. Ms_Rv0580c Induced the Hypoxia Factor and ER Stress in Macrophages 

**Figure 5:**
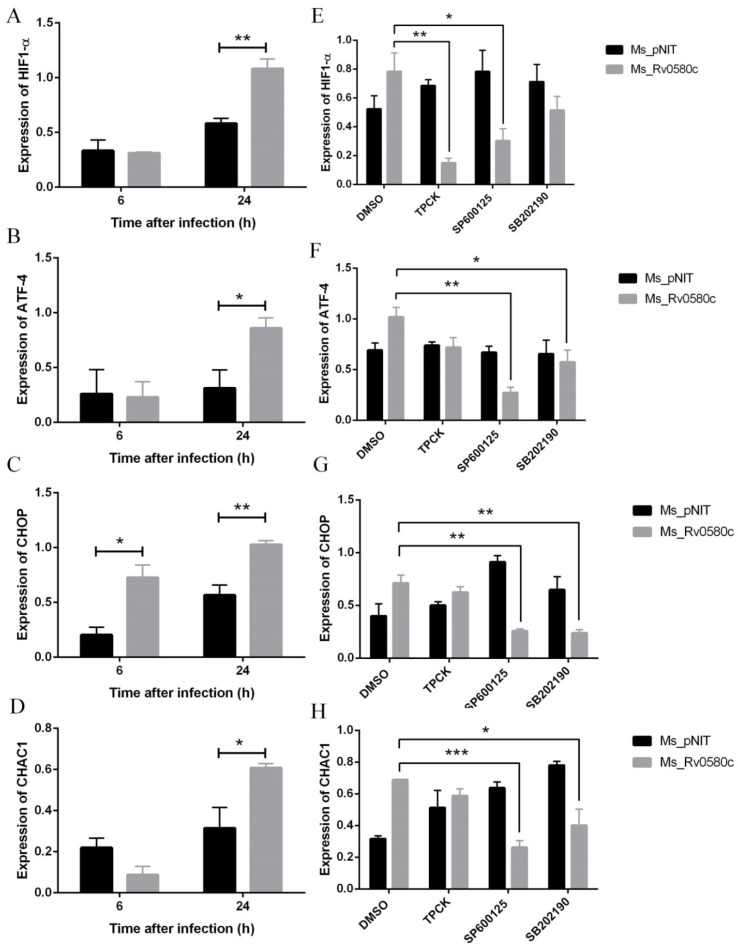
Rv0580c alters the expression of *HIF1-α* and ER stress maker genes in *M. smegmatis* infected macrophages. PMA- induced THP-1 cells were infected with Ms_pNIT and Ms_Rv0580c strains. At 6 h and 24 h post-infection, total RNAs were isolated and carried out the RT-PCR to determine the transcriptional levels of (**A**) *HIF1-α*, (**B**) *ATF-4*, (**C**) *CHOP* and (**D**) *CHAC1*. THP-1 cells were pre-treated with pharmacological inhibitors of NF-κB (TPCK), JNK (SP600125), and p38 (SB202190) and DMSO (vehicle control) at indicated concentrations, then infected with Ms_pNIT and Ms_Rv0580c. Total RNAs were extracted at 24 h post-infection and transcription levels of (**E**) *HIF1-α*, (**F**) *ATF-4*, (**G**) *CHOP*, and (**H**) *CHAC1* were determined by RT-PCR. The β-actin gene of macrophages was used as an internal control. Data points represent the mean of three separate experiments, and error bars are +/− SEM. * *p* < 0.05, ** *p* < 0.01, and *** *p* < 0.001.

IFN-γ-activated macrophages synergistically increase the immunosuppressive phenotypes and induce hypoxic condition in macrophages [30]. To explore whether Ms_Rv0580c can induce hypoxia, we examined the expression of hypoxia-inducible factor, HIF-1α, after infection of macrophages with recombinant strains. We detected the up-regulation of *HIF-1α* expression in Ms_Rv0580c infected THP-1 macrophages, as compared to Ms_pNIT infected THP-1 macrophages, at 6 h and 24 h post-infection (Figure 5A).

HIF-1α is responsible for the activation of endoplasmic reticulum (ER) stress [31]. To investigate whether Ms_Rv0580c up-regulated HIF-1α activates ER stress, we analyzed the expression of ER stress marker genes in recombinant strains infected THP-1 macrophages. Our results found that the expression of ER stress marker genes *ATF4*, *CHOP,* and *CHAC1* were up-regulated in Ms_Rv0580c infected THP-1 macrophages as compared to Ms_pNIT infected THP-1 macrophages, at 24 h post-infection (Figure 5B–D). Collectively, these results suggest that Ms_Rv0580c might exert its effect via modulating the HIF-1α activated ER stress.

### 2.9. Ms_Rv0580c Induced the Hypoxia Factor and ER Stress via NF-kB/JNK/p38 Axis

To examine whether the Ms_Rv0580c regulates the *HIF-1α* and ER stress marker genes, we pre-treated the THP-1 macrophages with TPCK (NF-κB inhibitor), SP600125 (JNK inhibitor) and SB202190 (p38 inhibitor), separately, for 1 h before infection with Ms_Rv0580c and Ms_pNIT. The results showed that after treatment with NF-κB inhibitor and JNK inhibitor, *HIF-1α* expression was down-regulated in Ms_Rv0580c infected THP-1 macrophages, as compared to Ms_pNIT infected THP-1 macrophages, at 24 h post-infection, while, after treatment with p38 inhibitor, *HIF-1α* expression was unaffected (Figure 5E). Moreover, *ATF4*, *CHOP,* and *CHAC1* expression were down-regulated after treatment with JNK and p38 inhibitors in Ms_Rv0580c infected THP-1 macrophages, as compared to Ms_pNIT infected THP-1 macrophages, at 24 h post-infection, while, after treatment with the NF-κB inhibitor, the expression of ER stress marker genes was unaffected (Figure 5F–H). These data implicated that the NF-κB/JNK axis participated in the Ms_Rv0580c-mediated expression of *HIF-1α*, while the JNK/p38 axis was involved in Ms_Rv0580c-mediated ER stress induction.

### 2.10. Ms_Rv0580c Induced Mitochondria-independent Apoptosis in Macrophages 

**Figure 6:**
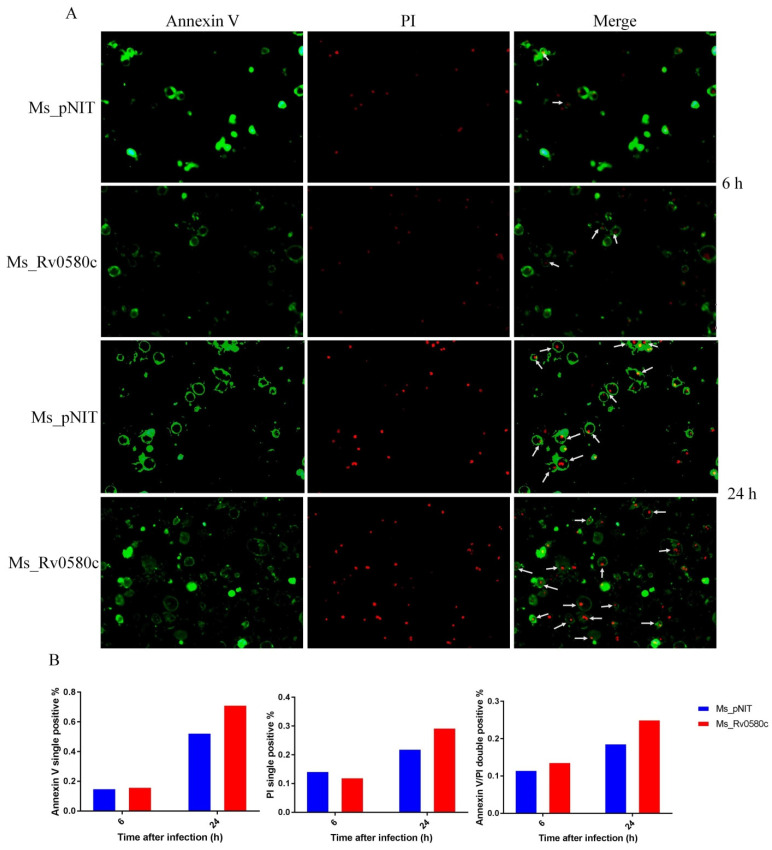
Rv0580c boosts the apoptosis in *M. smegmatis* infected macrophages. (**A**) Fluorescence micrographs of THP-1 macrophage cells, at indicated time points, of infected Ms_pNIT and Ms_Rv0580c. Macrophage monolayers were stained with both Annexin V-FITC and propidium iodide (PI) to visualize the early and late stages of apoptosis of THP-1 cells, respectively. The early apoptotic THP-1 cells with intact membranes are in green; later apoptotic cells that have already lost their membrane integrity are in red. (**B**) The relative content of Annexin V single positive, PI single positive, and Annexin V/PI double positive.

To investigate whether Ms_Rv0580c is involved in apoptosis, we performed the Annexin/PI double staining assay. The THP-1 macrophages were stained with Annexin V-FITC and propidium iodide (PI) at 6 h and 24 h post-infection with Ms_Rv0580c and Ms_pNIT. Annexin V-FITC stained the early apoptotic cells with green fluorescence, and PI stained the necrotic cells with red fluorescence, while both stained the late apoptotic cells. We observed that comparative fluorescence spots in Ms_Rv0580c infected THP-1 macrophages, V-FITC single positive and V-FITC/PI double positive were significantly higher, as compared to Ms_pNIT infected THP-1 macrophages (Figure 6A,B). To explore whether mitochondria are involved in Ms_Rv0580c-induced apoptosis, we examined the expression level of the mitochondria marker gene *Bnip3*, which is involved in mitochondria-dependent apoptosis [32]. Our results showed that there was no significant difference of the *Bnip3* expression level between Ms_Rv0580c and Ms_pNIT infected THP-1 macrophages (Figure S1). Collectively, these data suggested that Ms_Rv0580c can induce the macrophage apoptosis in mitochondria-independent manner.

## 3. Discussion

The *Rv0580c* gene encodes a conserved hypothetical protein in *M. tb* (https://mycobrowser.epfl.ch/genes/Rv0580c), whose function is yet to explore. The orthologs of Rv0580c are available in most genomes of the sequenced pathogenic mycobacteria, including *M. bovis*. Previously, Rv0580c protein was identified through the 1-D-SDS-PAGE method, extracted from the membrane protein fraction [33].

In this study, we evaluated the role of Rv0580c protein in the physiology and host interactions of mycobacteria. The mycobacterial colony morphology is a complex structure and directly correlated with virulence and metabolic adaptation [34]. Rv0580c shifted from smooth and non-wrinkled colony morphology to rough and wrinkled colony morphology in *M. smegmatis*, indicating the structural role of this protein. Alterations in mycobacterial cell wall are associated with susceptibility to the multiple stress responses that intracellular bacilli encounter within host macrophages. Increased sensitivity of Ms_Rv0580c to multiple stresses such as low pH, H_2_O_2_, SDS and antibiotics could be associated with cell wall alteration. This evidence was further supported by the increased cell wall permeability of Ms_Rv0580c, which increased the influx of compounds and drugs into the bacilli, leading to increase the sensitivity of Ms_Rv0580c to the anti-microbial compounds and antibiotics [35]. 

The expression of Rv0580c reduced the intracellular survival of *M. smegmatis* within THP-1 macrophages, while the in-vitro growth kinetics of *M. smegmatis* was unaffected by Rv0580c. The acidic environment and oxidative burst might be responsible for the reduced intracellular survival of Ms_Rv0580c inside macrophages [5]. Moreover, the fate of *M. tb* inside macrophages is directly associated with early host immune defense [36]. Interestingly, we found that Ms_Rv0580c up-regulated the IFN-γ expression and down-regulated the IL-10 expression in the macrophages, which activated the host immune defense [37]. The IFN-γ expression during early infection of *M. tuberculosis* [38] might be leads to suppress the intracellular survival of Ms_Rv0580c in macrophages [39]. NF-κB regulates the array of inflammatory cytokines gene expression, including IFN-γ and IL-10 [40]. We found that Ms_Rv0580c up-regulated IFN-γ expression via NF-κB and JNK signaling and down-regulated IL-10 expression via the NF-κB signaling pathway in macrophages. 

The *M. tb*-infected IFN-γ-activated macrophages increase the level of HIF-1α protein, to limits the pathogen [30]. HIF-1α is regulated via NF-κB to fight the infection [41]. JNK activation also regulates the expression of HIF-1α in hypoxic HeLa cells and fully-differentiated hypoxic neurons [42]. We found that Ms_Rv0580c up-regulated the *HIF-1α* expression in macrophages via the NF-κB/JNK axis, indicating the role of Rv0580c in the host defense against infection. 

Mycobacterial infection can disrupt the ER to prompt ER stress [43], which can be induced by HIF-1α via activation of the *ATF4*, *CHOP*, and *CHAC1* genes [31]. IFN-γ is the main inducer of ER stress [44,45]. The JNK/p38 signaling pathway is involved in ER stress and activates the ER stress responses [46]. We detected that Ms_Rv0580c up-regulated the expression of the ER stress marker genes *ATF4*, *CHOP*, and *CHAC1* via JNK/p38 signaling pathway, indicating the possible role Rv0580c in inducing ER stress in macrophages. Seimon et al. suggested that CHOP is also associated with ER stress-influenced apoptosis in the macrophages [47]. IFN-γ-induced apoptosis leads to the elimination of the intracellular bacterial pathogen, including *M. tb* [48,49]. Interestingly, we found that Ms_Rv0580c boosted macrophage apoptosis, might be correlated with the ER stress, leads to eliminate the intracellular bacilli from macrophages by inducing the host immune defense. Furthermore, Bnip3 protein is a target for the HIF-1α, which incites the permeability of inner and outer membranes of mitochondria, to activate mitochondrial-dependent cell death [31,50]. Ms_Rv0580c infected macrophages unchanged the expression of the *Bnip3* gene, suggesting that Rv0580c-induced the mitochondria-independent apoptosis in macrophages.

## 4. Materials and Methods

### 4.1. Bioinformatics Analysis

The nucleotide sequence and protein sequence of Rv0580c were downloaded from the *M. tb* H37Rv database (https://mycobrowser.epfl.ch/genes/Rv0580c). *M. tb* Rv0580c homologs, among different mycobacteria, were analyzed using the National Center for Biotechnology Information (NCBI) website (http://blast.ncbi.nlm.nih.gov). The sequence alignment of Rv0580c was performed using ClustalX with known proteins. The 3-dimensional model of Rv0580c was produced from the Protein Data Bank (PDB). To draw and display the structure-based sequence alignment, Espript 3 (http://espript.ibcp.fr/ESPript/cgi-bin/ESPript.cgi) was used. 

### 4.2. Bacterial Strains and Growth Conditions

*M. smegmatis* mc^2^155 strains were grown in Middlebrook (MB) 7H9 medium supplemented with 0.05 % tween 80 and 0.2 % glycerinum or MB 7H10 plates supplemented with 0.5 % glycerinum. Luria–Bertani (LB) medium was used to culture *E. coli* strains. Antibiotics were added at the following concentrations: ampicillin, 100 μg/mL; kanamycin, 50 μg/mL for *E. coli,* and 20 μg/mL for *M. smegmatis* mc^2^155. All cultures were incubated at 37 °C.

### 4.3. Detect Expression of Rv0580c in Recombinant M. smegmatis

The recombinant *M. smegmatis* mc^2^155 strain harboring myc-tagged Rv0580c (Ms_Rv0580c) and vector pNIT (Ms_pNIT) were cultured in MB 7H9 broth medium supplemented with 100 μg/mL kanamycin. At the OD_600_ value of 0.8, the recombinant strains were subjected to ε-caprolactam (inducer) for protein expression. Recombinant *M. smegmatis* cells fractionation was carried out essentially as described earlier [51], with minor modifications. In general, the recombinant strains Ms_pNIT and Ms_Rv0580c were harvested after 16 h inducer induction using centrifugation at a speed of 3000 × g for 10 min. The bacterial pellets were collected and washed by 1 × phosphate-buffered saline (PBS) three times. Samples were sonicated, then subjected to SDS-PAGE and detected by Western blotting with the mouse anti-myc antibody (TIANGEN, China). 

### 4.4. In-vitro Growth Assay 

To examine the growth kinetics of recombinant *M. smegmatis* strains, Ms_Rv0580c and Ms_pNIT strains were cultured triplicate in 50 mL 7H9 broth liquid medium supplemented with 0.05% (*v*/*v*) tween 80, without added antibiotics. The starting growths of both strains were equalized at OD_600_ 0.05 and cultured at 37 °C with continuous shaking. When the OD_600_ reached 0.8, then added the inducer and monitored the OD_600_ at 4 h of interval. The growth curves were plotted between OD_600_ and time intervals by using graph plotting software, GraphPad Prism 9.

### 4.5. Colony Morphology Assay

For colony morphology analysis, the recombinant strains Ms_Rv0580c and Ms_pNIT were cultured, when OD_600_ = 1 reached, then collected, washed, and re-suspended the culture in sterile 7H10 broth containing 2 % (*v*/*v*) inducer and incubated at 37 °C. Images of colonies were captured after 5 days. Colonies size and surface wrinkles were recorded, and diameters of the colonies were measured.

### 4.6. In-vitro Stress Assay

To analyze the impact of acidic stress on bacterial strains Ms_pNIT and Ms_Rv0580c, the pH gradient was generated by adding hydrochloric acid (HCl) into 7H9 liquid medium and sterilized by passing this medium through 2 µm filter. The bacterial strains were treated with low pH (pH 5) at 0, 3, 6, and 9 h time points. Then, bacterial cells were collected, ten-fold diluted and spotted on 7H9 solid media plates supplemented with kanamycin, at each time point. The survival percentages of bacteria were calculated after 3 days of incubation.

The effects of H_2_O_2_ and SDS on bacterial strains Ms_pNIT and Ms_Rv0580c were analyzed by disc diffusion assay. At mid exponential-phase, bacteria were collected, washed and mixed with 0.7% water agar and dappled on the 7H9 agar plates. After that, different concentrations (0.5%, 1.0%, and 2.0%) of 10 µl H_2_O_2_ were added on a 5.5 mm-diameter Whatman filter disc on the bacterial lawn, separately. Area of zone of inhibition was calculated after 3 days of incubation. Similarly, different concentrations (2.5%, 1.25%, and 0.625%) of 10 µl SDS were added on the 5.5 mm-diameter of Whatman filter disc on the bacterial lawn, separately, and calculated the area of zone of inhibition after 3 days of incubation. 

### 4.7. Antibiotics Sensitivity Assays

To analyze the sensitivity of antibiotics, ε-caprolactam induced Ms_pNIT and Ms_Rv0580c strains were cultured and exposed with four antibiotics (amikacin, vancomycin, erythromycin and roxithromycin) in this study. 

*Minimal inhibitory concentration (MIC)*. The MIC values were determined by 2-fold serial dilution of antibiotics in 7H9 liquid medium as previously delineated [23]. After 3 days of incubation at 37 °C, lowest concentration of antibiotics, at which the bacterial growth was inhibited, considered as MIC.

*Bactericidal capability test*. The ε-caprolactam induced bacterial strains were treated with 10×MIC of antibiotics, respectively. At 3 h, 6 h and 9 h post-treatment, bacterial cells were mottled on the 7H9 solid agar plates. After 3 days of incubation, bacterial numbers were counted and the survival percentage determined.

*Spot tests.* The culture of ε-caprolactam induced bacterial strains were grown up to OD_600_ 0.8. After that, 10× serial diluted the cultured bacteria and spotted on the 7H9 agar plates supplemented with antibiotics amikacin (5 µg/mL), vancomycin (7.5 µg/mL), erythromycin (25 µg/mL), and roxithromycin (50 µg/mL), separately. The bacteria were spotted on the 7H9 agar plate, considered as a control.

### 4.8. Cell Wall Permeability Assay

The ε-caprolactam-induced Ms_pNIT and Ms_Rv0580c strains were washed with 1× PBST buffer (1× PBS with 0.05% tween 80) and adjusted the OD_600_ 0.8. Subsequently, the bacterial cells (200 µl) were incubated with EtBr (2 μg/mL) (Sigma) or NR (20 μM) (Sigma) in a 96-well black fluoroplate. Immediately, the accumulation of EtBr or NR was measured at 544 nm (excitation) and 590 nm (emission) wavelengths by using the Synergy H1 Hybrid Microplate Reader, followed by 5 min intervals up to 55 min. For each well, all data were normalized to time zero reading. Experiments were reiterated triplet and obtained the similar results.

### 4.9. Intracellular Survival Assay 

The THP-1 human monocyte cells were seeded and maintained in RPMI 1640 medium added fetal bovine serum (FBS) (10%; *v*/*v*), penicillin (100 U/mL), streptomycin (100 U/mL) and L-glutamine (2 mM) (Invitrogen) at 37 °C in atmosphere containing 5% CO_2_. In addition, 1 × 10^6^ THP-1 cells were seeded in 12-well and 24-well tissue culture plates. The differentiation of THP-1 monocyte cell to macrophages were induced by the supplementation of Phorbol 12-myristate 13-acetate (PMA) (100 ng/mL) (Sigma) for 48 h before infection. 

The ε-caprolactam-induced Ms_pNIT and Ms_Rv0580c strains were grown for 16 h. After that, bacterial cells were washed 3 times with 1×PBS, then infected to the differentiated THP-1 macrophage cells, at MOI=10 (ratio between bacterial cells-to-THP-1 cells). After 4 h, the infected macrophage cells were washed three times by applying sterile 1× PBS to eliminate the extracellular bacilli, and added the fresh medium supplemented with hygromycin (100 μg/mL) and IVN (Sigma) (250 nM). At 6, 24, 48 and 72 h of post-infection, the infected macrophage cells were washed by 1× PBS and lysed by treated with SDS (0.05%; *w*/*v*). The cell lysates diluted serially 10-fold and mottled on the appropriate antibiotics supplemented 7H9 solid media. After 3 days of incubation, numbers of bacteria were counted to compute the intracellular survival of recombinant bacteria in macrophages. 

### 4.10. RNA Preparation and Real-time PCR (RT-PCR)

The PMA-differentiated THP-1 cells were infected with recombinant bacterial strains at MOI=10. At different time points of post-infection, total RNAs were isolated, as per the manufacturer’s protocol (TIANGEN, China). cDNA preparation was performed as per the manufacturer’s guidance (TIANGEN, China). RT-PCR amplification was performed by using Bio-Rad IQ5 with identical PCR condition: 5 min at 95 °C and 40 cycles for 30 s at 95 °C, 30 s at 60 °C, and 30 s at 72 °C. The relative expressions of mRNA of genes were calculated using respective primers (Table S2). The β-actin applied as internal control.

### 4.11. Cell Signaling Assay

All pharmacological inhibitors were obtained from Sigma, dissolved in Dimethyl sulfoxide (DMSO) (Sigma), and used at the following concentrations: 10 μM p38 signaling inhibitor SB202190 [29], 25 μM JNK signaling inhibitor SP600125 [28], and 30 μM NF-κB signaling inhibitor TPCK [27]. 0.1% (*v*/*v*) DMSO was added to the cultures as vehicle control. After 24 h post-infection, total RNAs were isolated and converted into cDNA, then carried out the RT-PCR to detect the mRNA level of cytokines by using specific primers (Table S2).

### 4.12. Apoptosis Assay

The THP-1 macrophage cells (2 × 10^6^ cells/ well) were infected with recombinant strains Ms_Rv0580c and Ms_pNIT, at MOI of 10. After 24 h post-infection, THP-1 cells were rinsed with 1×PBS three times, and Annexin V binding buffer (400 µl) was supplemented to the wells. After that, Annexin V-FITC (5 µl) and PI (10 µl) (Beibo, Shanghai, China) were added to the wells and incubated for 10 min in dark, at room temperature. The stained cells were examined and calculated, respectively, by employing fluorescence microscopy. The untreated cells were used as a negative control.

### 4.13. Statistical Analysis

All experiments were reiterated three times. The D’Agostino-Pearson normality test has been applied to normalize the data, and significant differences (*p*-value) between experimental group and control group were determined by unpaired Student’s t-test. Data points represent the mean of three separate experiments, and error bars are +/− SEM. *p*-values less than 0.05 were considered as significant, * *p* < 0.05, ** *p* < 0.01, and *** *p* < 0.001. Graphpad Prism 9 software was used for all statistical analysis and graphical representations.

## 5. Conclusions

**Figure 7:**
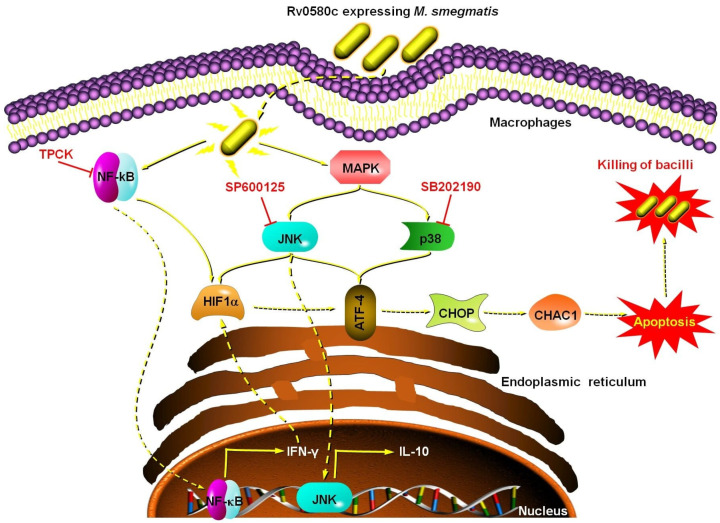
Schematic summary of Rv0580c expressing *M. smegmatis* interaction with macrophages. Rv0580c expressing *M. smegmatis* internalize by the host macrophage cell, where it activates the NF-κB and p38/JNK signaling pathways. NF-κB signaling activates the transcription of IFN-γ and HIF1-α. The p38/JNK signaling activates the ER stress marker genes possibly activate the ER stress, which leads to a boost in the apoptosis and kills the intracellular bacilli. TPCK (NF-κB inhibitor); SP600125 (JNK inhibitor); SB202190 (p38 inhibitor).

The Rv0580c protein is conserved in the pathogenic mycobacterium strains. The expression of the *Rv0580c* gene in *M. smegmatis* modified the colony architecture, and increased the sensitivity of *M. smegmatis* to the multiple stresses and antibiotics, by increasing the cell wall permeability. The intracellular survival of Ms_Rv0580c was reduced, which might be due to oxidative burst in macrophages. Ms_Rv0580c was up-regulated the IFN-γ via NF-κB/JNK signaling, and down-regulated the IL-10 via NF-κB signaling in the macrophages. Moreover, Ms_Rv0580c up-regulated the expression of *HIF-1α* via the NF-κB/JNK axis and the expression of ER stress marker genes via JNK/p38 axis in macrophages, as well as boosted the macrophages apoptosis, independent to the mitochondria (Figure 7). Our finding proposed that Rv0580c protein might be a considerable candidate having good potential for the development of a powerful vaccine against mycobacterium infection. 

## Data Availability

Not applicable.

## Supplementary Materials

The following are available online at https://www.mdpi.com/2076-0817/10/2/143/s1, Figure S1: Rv0580c unchanged the expression of BinP, Table S1: Homology percentage of Rv0580c amino acids sequences with its mycobacterium orthologous by BlastP, Table S2: Used primers in this study, Table S3: MIC values of antibiotics.
