# Supplementary material for: Mycobacterium tuberculosis Rv0580c Impedes the Intracellular Survival of Recombinant Mycobacteria, Manipulates the Cytokines, and Induces ER Stress and Apoptosis in Host Macrophages via NF-κB and p38/JNK Signaling
Source: Pathogens. 2021 Feb 1;10(2):143. doi: 10.3390/pathogens10020143 (PMC7912736; doi:10.3390/pathogens10020143)
Supplement: Supplementary file 1 [file pathogens-10-00143-s001.pdf]

Supplementary materials

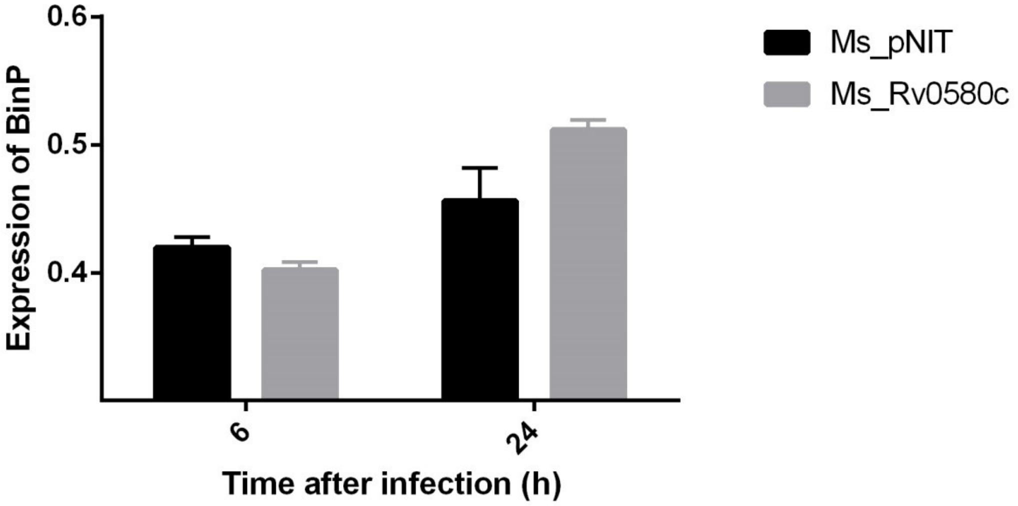

Figure S1. Rv0580c unchanged the expression of BinP. PMA- induced THP-1 cells were infected with Ms_pNIT and Ms_Rv0580c strains. 6 h and 24 h post infection, total RNAs were isolated and performed the RT-PCR to determine the transcriptional level of BinP. β-actin gene of macrophages THP-1 cells was used as an internal control.

Table S1. Homology percentage of Rv0580c amino acids sequences with its mycobacterium orthologous by BlastP.

Strains	Accession No.	Identity (%)
M. tuberculosis str. Haarlem	EBA41097.2	100.00
M. tuberculosis CDC1551	AAK44833.1	100.00
M. tuberculosis SUMu003	EFP20741.1	100.00
M. bovis	CEJ32381.1	99.39
M. canettii	WP_014000309.1	99.39
M. tuberculosis GuangZ0019	EQM23392.1	99.23
M. malmoense	WP_083011943.1	78.12
M. heidelbergense	WP_083074017.1	77.50
M. kansasii	WP_023370711.1	76.88
M. bohemicum	WP_085179266.1	76.43
M. gastri	WP_036411571.1	76.25
M. gastri 'Wayne'	ETW25170.1	76.25
M. haemophilum	WP_054880698.1	75.78
M. riyadhense	WP_085249187.1	75.62
M. sp. ACS4054	WP_067902803.1	74.53

Table S2. Used primers in this study.

Primers	Sequence (5'-3')
pNIT-Rv0580c-F	CGCCATATGGGATCTCGGTGAAG
pNIT-Rv0580c-R	CTACGAATTGTCGCGGATCCGGC
IFN-γ-F	GAAGAATTGGAAAGAGGAGAG
IFN-γ-R	TGGATGCTCTGGTCATCTTT
IL-10-F	ACCTGGGTTGCCAAGCCTTGT
IL-10-R	GCTCCACGGCCTTGCTCTTGTTT
HIF1-α-F	CTCAAAGTCGGACAGCCTCA
HIF1-α-R	CCCTGCAGTAGGTTTCTGCT
ATF-4-F	GTCAGTCCCTCCAACAACA
ATF-4-R	GGTGTCTTCCTCCTTTATGC
CHOP-F	GCACCTCCCAGAGCCCTCACTCTCC
CHOP-R	GTCTACTCCAAGCCTTCCCCCTGCG
CHAC1-F	CCTGAAGTACCTGAATGTGCGAGA

CHAC1-R	GCAGCAAGTATTCAAGGTTGTGGC
Bnip3-F	GGATGCAGGAGGAGAGCCT
Bnip3-R	CGAG GTGGGCTGTCACAGT
β-actin-F	GTGACGTTGACATCCGTAAAGA
β-actin-R	TGTGAGTCCCGGAGCGTGCAGTT

Table S3. MIC values of antibiotics.

**Antibiotics**	**MIC (μg/ml)**	
	Ms_Rv0580c	Ms_pNIT
Vancomycin	15.625	7.812
Amakine	3.906	1.953
Roxithromycin	250.0	125.0
Erythromycin	125.0	31.25
